# Design, Synthesis, and Anti-Proliferative Evaluation of [1,1′-biphenyl]-4-ols as Inhibitor of HUVEC Migration and Tube Formation

**DOI:** 10.3390/molecules17078091

**Published:** 2012-07-05

**Authors:** Yan Ran, Liang Ma, Xuewei Wang, Jinying Chen, Guangcheng Wang, Aihua Peng, Lijuan Chen

**Affiliations:** 1 Pharmacy College of West China of Sichuan University, Chengdu, Sichuan 610065, China; 2 State Key Laboratory of Biotherapy, West China Hospital, West China Medical School, Sichuan University, Chengdu, Sichuan 610041, China

**Keywords:** biphenol, endothelial cell, anti-proliferative, migration, tube formation

## Abstract

Allylated biphenol neolignans contain a variety of chemopreventive entities that have been used as anti-tumor drug leads. Herein, 37 allylated biphenols were evaluated for anti-proliferative activity by the MTT assay and inhibitory effect on the migration and tube formation of HUVECs featuring anti-angiogenic properties. 3-(2-Methylbut-3-en-2-yl)-3′,5′-bis(trifluoromethyl)-[1,1′-biphenyl]-4-ol (**5c**) exerted an inhibitory effect on HUVECs compared to honokiol (IC_50_ = 47.0 *vs.* 52.6 μM) and showed significant blocking effects on the proliferation of C26, Hela, K562, A549, and HepG2 (IC_50_ = 15.0, 25.0, 21.2, 29.5, and 13.0 μM, respectively), superior to those of honokiol (IC_50_ = 65.1, 62.0, 42.0, 75.0, and 55.4 μM, respectively). Importantly, compound **5c** inhibited the migration and capillary-like tube formation of HUVECs *in vitro*.

## 1. Introduction

Angiogenesis is a complicated multistep process involving endothelial cell (EC) activation, invasion, migration, proliferation, tube formation, and finally capillary network formation in several solid tumors and haematological malignancies [1]. Tumor growth depends on the recruitment of new blood vessels from pre-existing vasculature. Without the development and progression of new blood vessels, tumors cannot deteriorate beyond a critical size or metastasize to other organs [2,3,4].

Endothelial cells play crucial roles in a series of physiological processes (e.g., wound healing, reproduction, and embryonic development) and pathophysiological events (e.g., solid tumor growth, psoriasis, and diabetic retinopathy). The well-established target of anti-angiogenesis is genetically stable, non-transformed ECs, which are less prone to acquire drug resistance [5,6]. When tumor cells secrete pro-angiogenic growth factors that bind to receptors on dormant ECs, leading to ECs activation, stimulation, vasodilatation and an up-regulation of vessel permeability, the activated ECs rapidly detach from the extracellular matrix and basement membrane by secreting proteases (matrix metalloproteinases). Subsequently, they migrate and proliferate to sprout and self-assemble into new branches from the pre-existing vasculatures [7,8,9]. Therefore, suppression of EC activation, invasion, migration, proliferation, tube formation, and finally capillary network formation seem to be an effective and relevant strategy for blocking tumor progression and cancer development. Up to now, more than 80 anti-angiogenic drugs are currently in clinical trials. Notably, many angiogenesis inhibitors have been discovered in natural resources, such as fungi, mushrooms, shark muscle and cartilage, sea corals, green tea, ginseng, and garlic by screening of EC cultures [10].

Biphenols are widely found in naturally products, including lignans, flavonoids, tannins, together with coumarins, peptides, glycopeptides, *etc*. [11]. Natural biphenol products contained a range of chemo-therapeutic and chemo-preventive entities that have the capacity of preventing and inhibiting the development of malignancies [12,13,14]. However, only a few studies have focused on the structural modification and structure-activity relationship (SAR) of biphenols targeting angiogenesis or cancer [15,16]. Disconnections and chemical synthesis have been undertaken to develop potent allylated biphenols to improve biological activity or clarify the SAR in our previous study [17].

In this study, 37 allylated biphenols were designed, synthesized, and subsequently screened. Among our synthetic compounds, 3-(2-methylbut-3-en-2-yl)-3′,5′-bis(trifluoromethyl)-[1,1′-biphenyl]-4-ol (**5c**) exhibited a medium inhibitory effect on human umbilical vein endothelial cells (HUVECs; IC_50_ = 40.0 μM) in contrast to honokiol (IC_50_ = 57.0 μM) and blocked the proliferation of C26 (murine colon adenocarcinoma, IC_50_ = 15.0 μM), Hela (human cervical carcinoma, IC_50_ = 25.0 μM), K562 (human erythromyeloblastoid leukemia; IC_50_ = 21.2 μM), A549 (human lung carcinoma; IC_50_ = 29.5 μM), and HepG2 (human hepatocellular liver carcinoma; IC_50_ = 13.0 μM) showing superior activity to honokiol (IC_50_ = 65.1, 62.0, 42.0, 75.0, and 55.4 μM, respectively). Importantly, **5c** showed much more potent inhibitory potency against the migration and tube formation of HUVECs than those of honokiol. The anti-proliferative activity of allylated biphenols led to the establishment of a structure-activity relationship. In contrast to our previous report, compound **5c** exhibited comparable inhibitory activity to honokiol, but less potent *in vitro* inhibitory potency than 3,5′-diformalhonokiol (compound **8c** in reference [17]) against HUVECs, A549, and, HepG2. Although compound **5c** could significantly suppress HUVEC migration and tube formation, **5c** was not found to prevent the newly-grown segmental vessels from the dorsal aorta of zebrafish, and inappropriate vascularisation in the transgenic zebrafish screening model.

## 2. Results and Discussion

### 2.1. Chemistry

**Scheme 1:**
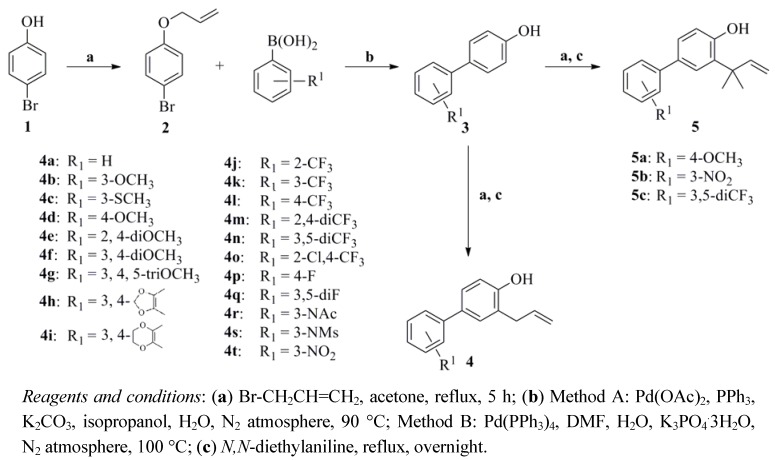
Synthesis of compounds **4a–t** and **5a–c**.

As depicted in Scheme 1, 1-allyloxy-4-bromobenzene (**2**) was prepared in a satisfactory yield (95%) by a convenient procedure starting from commercially available *p*-bromophenol and employing 1.1 equiv of allyl bromide in the presence of anhydrous K_2_CO_3_ (1.3 equiv) as base and acetone as solvent. The intermediates **3a–t** were obtained through Suzuki-Miyaura reactions [17,18]. Two cross-coupling methods were developed for the condensation of appropriate arylboronic acids with **2** under palladium catalysis under a N_2_ atmosphere. Method A involved the use of Pd(OAc)_2_ as a cross-coupling catalyst, PPh_3_ as a reducing agent and K_2_CO_3_ (2.0 M) as a base in isopropanol as solvent. The intermediates **3a–i** was obtained by Method A in 55–80% yields. Another coupling method (used for **3j–t**) was carried out by using Pd(PPh_3_)_4_ as a catalyst, 2.0 M K_3_PO_4_·3H_2_O as a base and DMF as solvent. The intermediates **3j–t** were obtained by Method B in much better yields (75–90%).

**Scheme 2:**
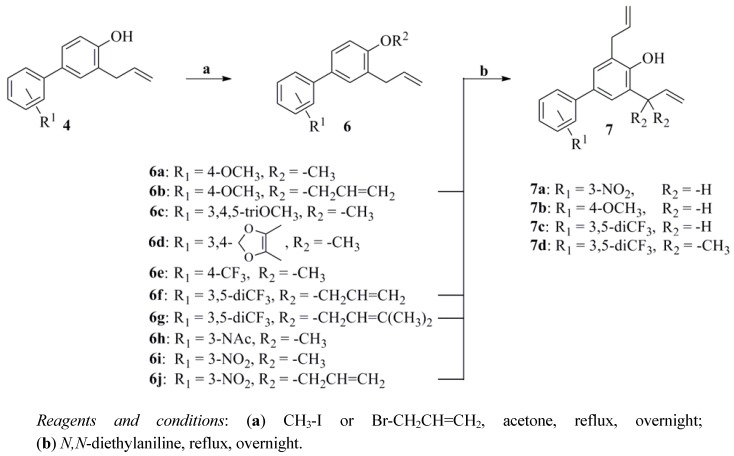
Synthesis of compounds **6a–j**, and **7a–d**.

The O-allylation of **3** with 3-bromoprop-1-ene or 1-bromo-3-methylbut-2-ene was performed in a good yield in the presence of anhydrous K_2_CO_3_. Next, the corresponding compounds **4a–t** and **5a–c** were synthesized through the Claisen rearrangement, which belongs to the [3,3]-sigmatropic concerted rearrangement category, in *N,N*-diethylaniline (boiling point = 216 °C) as solvent [19,20]. Moreover, the other allylated biphenols **6a–j** and **7a–d** were prepared following similar synthetic methods (Scheme 2).

### 2.2. Anti-Proliferative Activity and SAR Study

In order to find new potential angiogenic inhibitors, 37 allylated biphenols were primarily investigated for anti-proliferative activity on C26 and Hela tumor cells by the MTT assay and these results are shown in Table 1. In addition, predicted octanol/water log P (Clog P, miLog P, and Xlog P) were calculated using the *ChemDraw software*, *Molinspiration* online service and *XlogP3* online service, respectively, to provide a measure of lipophilicity. Honokiol, a potent anti-angiogenic and anti-tumor drug lead, was selected as a positive control.

As for the twenty 3-allyl-[1,1′-biphenyl]-4-ols **4a–t**, the preliminary IC_50_ values indicated that only six substituted derivatives (*i.e.*, 3′-SCH_3_ in **4c**, 2′-CF_3_ in **4j**, 2′,4′-diCF_3_ in **4m**, 3′,5′-diCF_3_ in **4n**, 2′-Cl,4′-CF_3_ in **4o**, and 3′-NO_2_ in **4t**) exhibited comparable or superior activity to honokiol (65.1 μM for C26, and 62.0 μM for Hela) on anti-proliferative activity, with **4n** being the most efficient (34.5 μM for C26, and 36.0 μM for Hela). Importantly, **4n** achieved an approximately 2.0-fold improvement in inhibitory activities against C26 and Hela compared to those of honokiol. With respect to all the O-alkylated biphenyls (compounds **6a–j**), the introduction of a methyl or allyl group did not provide any advantage for the anti-proliferative effects. Although O-methylation of **4g** (3′,4′,5′-triOCH_3_) led to the corresponding derivative **6c**, which showed weak inhibitory activity (76.0 μM for C26), it still was inactive in blocking the proliferation of Hela cells (>100.0 μM). Inspection of the structural features of **4a–t**, and **6a–j** and activity indicated that the phenolic hydroxyl group at the C-4 position of the biphenyl motif was very essential for anti-proliferation activity because the O-alkylated compounds were inactive (e.g., **4n** *vs.* **6f**; 4**t** *vs.* **6i**) and the number of the trifluoromethyl substituents potentially affected the inhibitory potency (e.g., one CF3 in **4j** *vs*. two CF3 in **4n**).

**Table 1:** IC_50_ values against C26 and Hela cells and calculated properties of allylated biphenols.

Compd	MW ^a^	cLogP ^b^	miLogP ^c^	xLogP ^d^	IC_50_ (μM)		
					C26	Hela	K562
**Honokiol**	266.33	5.03	5.01	4.98	65.1	62.0	42.0
**4a**	210.10	4.37	4.47	4.26	69.0	96.0	NI ^e^
**4b**	240.12	4.25	4.51	4.23	>100.0	82.0	NI
**4c**	256.36	4.81	4.88	4.78	63.0	62.0	71.2
**4d**	240.30	4.21	4.53	4.23	67.0	81.5	NI
**4e**	270.03	4.12	4.51	4.21	96.0	58.0	90.0
**4f**	270.13	4.12	4.12	4.21	90.0	>100.0	NI
**4g**	300.35	3.99	4.10	4.18	74.0	95.5	110.5
**4h**	254.09	4.15	4.36	4.08	80.0	89.5	NI
**4i**	268.31	3.88	3.98	3.98	>100.0	76.5	NI
**4j**	278.27	5.29	5.32	5.15	53.1	52.3	41.6
**4k**	278.09	5.29	5.34	5.15	54.0	70.1	61.5
**4l**	278.09	5.29	5.37	5.15	61.7	68.5	55.8
**4m**	346.08	6.21	6.19	6.03	45.1	48.7	65.8
**4n**	346.08	6.21	6.19	6.03	34.5	36.0	56.3
**4o**	312.05	5.85	5.97	5.78	59.5	50.0	62.4
**4p**	228.26	4.53	4.64	4.36	57.1	94.5	82.0
**4q**	246.25	4.69	4.73	4.46	88.1	72.5	78.0
**4r**	267.13	3.28	3.67	3.44	67.2	>100.0	NI
**4s**	303.09	2.59	3.68	3.23	>100.0	80.0	NI
**4t**	255.09	3.95	4.41	4.09	48.5	56.5	71.0
**5a**	268.35	5.05	5.50	5.11	74.0	95.0	80.0
**5b**	283.12	4.96	5.38	4.97	58.0	55.0	53.0
**5c**	374.32	7.01	7.16	6.91	15.0	25.0	21.2
**6a**	254.32	4.51	4.60	4.56	>100.0	>100.0	NI
**6b**	280.15	5.20	5.24	5.20	>100.0	>100.0	NI
**6c**	314.15	4.26	4.17	4.50	76.0	>100.0	NI
**6d**	268.11	4.41	4.43	4.40	>100.0	>100.0	NI
**6e**	292.11	5.56	5.44	5.47	>100.0	>100.0	NI
**6f**	386.11	7.17	6.90	7.00	>100.0	>100.0	NI
**6g**	414.14	7.17	7.94	7.86	>100.0	>100.0	NI
**6h**	281.35	3.54	3.74	3.77	>100.0	>100.0	58.0
**6i**	269.11	4.47	4.48	4.42	>100.0	>100.0	NI
**6j**	295.12	5.03	5.12	5.06	>100.0	>100.0	NI
**7a**	295.12	5.04	4.95	5.16	44.7	51.3	55.2
**7b**	280.15	5.30	5.07	5.31	65.0	72.0	73.4
**7c**	386.33	7.27	6.74	7.10	25.5	36.0	37.3
**7d**	414.38	8.07	7.71	7.98	29.0	40.0	42.1

^a^ Molecular weight; ^b^ Calculated by ChemDraw Ultra, version 10.0; ^c^ Calculated by Molinspiration online service; ^d^ Calculated by XLOGP3 online service; ^e^ no inhibition.

In order to explore the SAR information based on the aforementioned analysis, we further synthesized compounds **5a–c**, in which the allyl group at the C-3 site was replaced by a 2-methylbut-3-en-2-yl substituent, and **7a–d** with the introduction of one more allyl (compounds **7a–c**) or a 2-methylbut-3-en-2-yl (**7d**) group at the C-5 position of the biphenol scaffold. Interestingly, 3-(2-methylbut-3-en-2-yl)-3′,5′-bis(trifluoromethyl)-[1,1′-biphenyl]-4-ol (**5c**) was found to exhibit more potent inhibitory activity against C26 and Hela tumor cells (IC50 = 15.0, and 25.0 μM, respectively) than those of honokiol and the chemical entity also contained the two trifluoromethyl (CF_3_) substituents. However, the further modification of **5c** that led to **7c** and **7d** didn’t achieve an effective improvement in inhibitory potency compared to honokiol, indicating that the best number of allyl or 2-methylbut-3-en-2-yl moieties in the same phenyl ring seemed to be less than two, as well as highlighting that the introduction of allyl or 2-methylbut-3-en-2-yl groups at the C-5 position of the biphenol was forbidden.

A relevant strategy for anti-angiogenesis is effectively inhibition of the proliferation of ECs. Thus, the result of anti-proliferative activity against HUVECs was selected as the main index and the IC_50_ values against A549 and HepG2 cells were also outlined in Table 2. With comparable or superior inhibitory potency against C26 and Hela cells compared to honokiol, nine allylated biphenols were next selected for biological evaluation, with **5c** being the most efficient. Compound **5c** exhibited a moderate inhibitory activity against the proliferation of HUVECs (IC_50_ = 57.0 μM) in contrast to honokiol (IC_50_ = 40.0 μM) and exerted remarkable cytotoxic activities against A549 and HepG2 cells (IC_50_ = 29.5 and 13.0 μM, respectively). At the same time, **7c** presented comparable inhibitory potencies. The anti-proliferative effects on HUVECs and the inhibitory activities against A549 and HepG2 of tested compounds showed consistent tendencies. In addition, there was a potential positive correlation between the lipophilicity (Log P and cLogP) and inhibitory potency.

**Table 2:** IC_50_ values against HUVECs and A549, HepG2 cells of the selected allylated biphenols.

Compd.	IC_50_ (μM)		
	HUVEC	A549	HepG2
**Honokiol**	40.0	75.0	55.4
**4j**	82.0	59.7	52.4
**4k**	76.0	73.0	58.3
**4l**	74.0	76.0	60.3
**4n**	>100.0	50.0	37.5
**4o**	>100.0	72.0	68.0
**5b**	77.0	64.0	56.0
**5c**	47.0	29.5	13.0
**7c**	48.0	30.0	26.5
**7d**	70.0	32.0	33.0

### 2.3. Effects on HUVEC Migration

**Figure 1:**
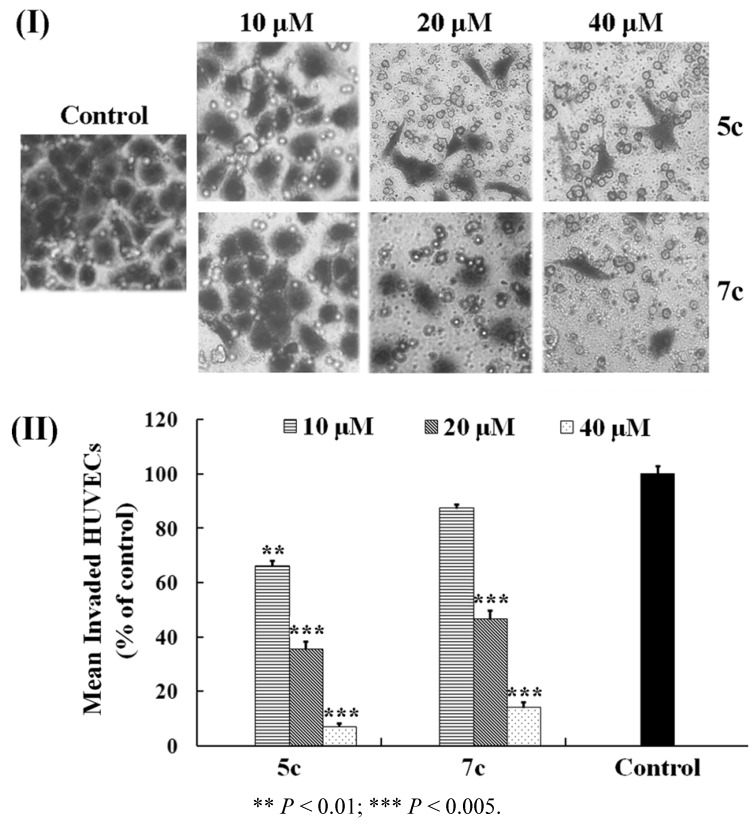
Effects of **5c** and **7c** on the HUVECs migration. (**I**) HUVECs seeded in 24-well Chambers were incubated for 6 h with medium alone (control) or contained the indicated concentration (10, 20, and 40 μM) of **5c** and **7c**. The photographs exhibited the migrated cells on the lower surface of the filter stained with 1% crystal violet under a phase contrast microscopy (magnification: 200×). (**II**) Mean invaded HUVECs (% of control) werequantitatively analyzed by the inhibitory effects of **5c** and **7c**. Data represented the mean ± standard error (SE) from three independent experiments.

EC migration is a relevant process in chemotaxis and an indispensable step to form new blood vessels. Inhibition on the process could block the formation of new blood vessels and further suppress the development of cancer. Therefore, to characterize the effects of allylated biphenols **5c** and **7c** on HUVEC migration, an *in vitro* migration assay was performed by the application of a slightly modified Boyden chamber. As depicted in Figure 1I, the HUVECs actively migrated to the serum-containing lower chamber within 6 h under the untreated conditions (control). Compared to the control, the mean number of invaded HUVECs (% of control group) treated with **5c** and **7c** at the concentration of 40 μM were 7.1 and 14.2%, respectively, and the cell migration was not likely to occur [Figure 1(II)]. After treatment with **5c** and **7c**, even at 20 μM, the inhibitory effects on cell migration were also effective and the mean invasion rates remained below 50.0%. However, at 10 μM, the two biphenols hardly inhibited HUVEC migration. As a consequence, **5c** and **7c** exerted the potent inhibitory activity on the migration of HUVECs in a concentration-dependent manner and **5c** was more potent than **7c**.

**Figure 2:**
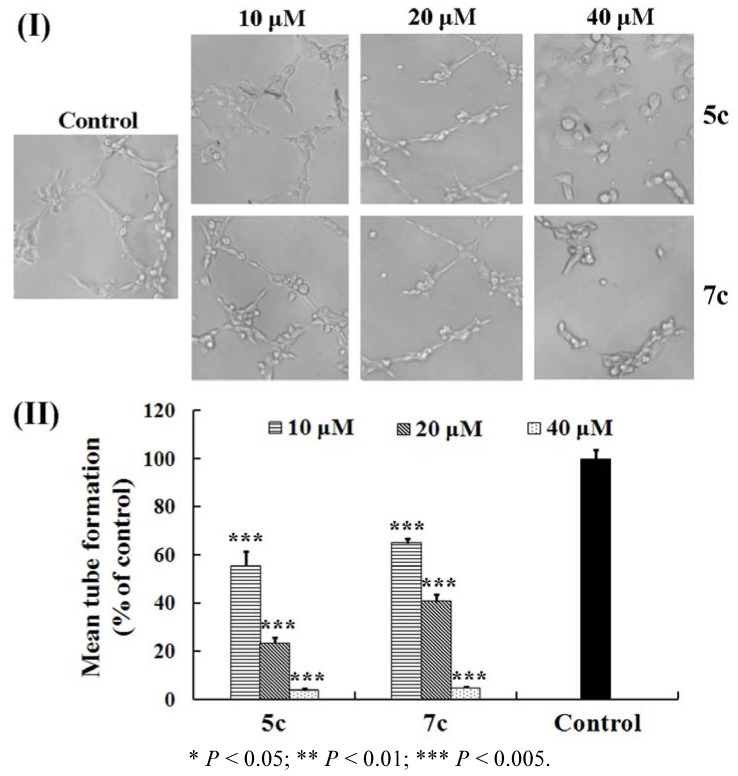
Effects of **5c** and **7c** on the HUVECs Tube Formation. (**I**) HUVECs (1 × 10^4^ cells) suspended in DMEM containing the tested compound (10, 20, and 40 μM) were added to the Matrigel. Control was treated with DMEM alone. After incubation for 24 hours at 37 °C, capillary networks were photographed and quantified (magnification: 200×). (**II**) The number of mean tube formation was counted in five randomly chosen regions and expressed as the percentage of the control. The results were expressed as mean ± SE.

In the later stages of angiogenesis, ECs will assemble into an interconnected tubular network which is similar to *in vivo* capillary vascular beds. Inhibition on this formation of capillary-like tube networks will block the formation of new blood vessels. A tube formation assay was performed by plating HUVECs on Matrigel. In the blank control, the cells exhibited high mobility on Matrigel and constructed an intact tube network in 24 hours [Figure 2(I)]. Compared to the control, the mean number of tube formation (% of control) treated with **5c** and **7c** at the concentration of 40 μM were 3.7 and 4.7%, respectively, and the disrupted tubular structures were sparse and incomplete [Figure 2(II)]. After the treatment with **5c** and **7c**, even at 20 μM, the inhibitory effects were also effective and the mean tube formation remained at 23.4 and 40.6%, respectively. However, served at the low concentration of 10 μM, the two compounds were totally unable to inhibit tube formation of HUVECs. Our observation indicated that allylated biphenols (**5c** and **7c**) could effectively terminate the formation of capillary-like tube networks and the effect of **5c** was superior to that of **7c** at the same concentration.

## 3. Experimental

### 3.1. Chemistry

Chemical reagents of analytical grade were purchased from Chengdu Changzheng Chemical Factory (Sichuan, China). ^1^H-NMR spectra were recorded at 400 MHz on a Varian Gemini 400 spectrometer (Varian, Palo Alto, CA, USA) and are reported in parts per million. Chemical shifts (δ) are quoted in ppm relative to the internal standard tetramethylsilane (TMS), where (δ) TMS = 0.00 ppm. The multiplicity of the signals is indicated as s, singlet; d, doublet; t, triplet; q, quartet; and m, multiplet defined as all multipeak signals where overlap or complex coupling of signals makes definitive descriptions of peaks difficult. Mass spectra were measured by a Premier quadrupole-time of flight (Q-TOF) mass spectrometer (Micromass, Manchester, UK) utilizing electrospray ionization (ESI). The purity of compounds was determined to be ≥97% by HPLC analysis using a photodiode array detector (Waters, Milford, MA, USA) and the chromatographic column was an Atlantis C_18_ (150 mm × 4.6 mm, i.d. 5 μm) (Waters, Milford, Ireland). All compounds were dissolved as 0.1 mg/mL solutions in HPLC quality methanol with 10 μL injected on a partial loop fill at a flow rate of 1 mL/min and the column chamber was kept at 20 °C for the analysis. The moble phases were 70% methanol and 30% water (0.1% formic acid).

#### 3.1.1. General Procedure Step I for the Preparation of 1-Allyloxy-4-Bromobenzene (**2**)

Allyl bromide (1.9 mL, 22 mmol) was slowly added into a solution of *p*-bromophenol (3.46 g, 20 mmol) and anhydrous K_2_CO_3_ (3.6 g, 26 mmol) in acetone (25 mL), and the mixture was refluxed for 5 hours (TLC monitoring). After completing and cooling, the mixture was filtered to remove the solid and the filtrate was evaporated to dryness. The residue was extracted with diethyl ether (20 mL × 3) and 10% NaOH (20 mL × 2) and then the organic layer was combined and washed by brine (20 mL × 2), dried over anhydrous MgSO_4_, and concentrated under reduced pressure to afford a colorless oil (4.05 g, 19.01 mmol, 95.0%).

#### 3.1.2. Step II for the Preparation of Intermediates **3** by Suzuki-Coupling Reaction

*Method A* (for **3a-i**): Compound **2** (1.0 mmol) and arylboronic acids (1.2 mmol) were dissolved in isopropanol (4 mL) at room temperature and stirred for 10 min. After a clear solution was formed, Pd(OAc)_2_ (0.01 mmol), PPh_3_ (0.03 mmol) and anhydrous K_2_CO_3_ (2.0 mol/L, 1 mL) were quickly added under a N_2_ atmosphere, and the resulting mixture was stirred for a further 18 hours at 90 °C (TLC monitoring). After completion of the reaction, the mixture was filtered, and extracted with ethyl acetate (10 mL × 3). The extracts were combined and washed with brine (10 mL × 2), dried over anhydrous MgSO_4_, and concentrated. The residue was purified by silica gel column chromatography (ethyl acetate/petroleum ether = 1:10) to give the intermediates **3a–i** in satisfactory yields (55–80%).

*Method B* (for **3j–t**): Compound **2** (1.0 mmol) and arylboronic acids (1.2 mmol) were dissolved in DMF (4 mL) at room temperature and stirred for 5 min. After a clear solution was formed, Pd(PPh_3_)_4_ (0.01 mmol), and a solution of K_3_PO_4_·3H_2_O (2.0 mmol) in water (1 mL) were quickly added under a N_2_ atmosphere, and the mixture was heated for 18 h at 100 °C (TLC monitoring). After completion, the reaction mixture was filtered, and extracted with ethyl acetate (10 mL × 3). The extract was combined and washed with brine (10 mL × 2), dried over anhydrous MgSO_4_, and concentrated under reduced pressure. The residue was purified by gel chromatography (ethyl acetate/petroleum ether = 1:10) to give the intermediates **3j–t** in satisfactory yields (75–90%).

#### 3.1.3. Step III for the Preparation of **4a–t**

Allyl bromide or 1-bromo-3-methylbut-2-ene (1.3 mmol) was added to the solution of **3** (1.0 mmol) and anhydrous K_2_CO_3_ (2.0 mmol) and refluxed for 5 hours. After completion (TLC monitoring) and cooling, the mixture was filtered, and the solution was evaporated to dryness. Next, the crude product was dissolved in *N,N*-diethylaniline and refluxed for 10 hours under a N_2_ atmosphere (TLC monitoring). After completion and cooling, the solution was adjusted to pH = 4 with 2.5 N HCl and extracted with ethyl acetate (10 mL × 2) and the organic layer was combined and washed with water (10 mL × 2) and brine (10 mL × 1), dried over anhydrous MgSO_4_, and concentrated under reduced pressure. The residue was purified by silica gel column chromatography (ethyl acetate/petroleum ether = 1:10) to give the targeted **4a–t**. The yield represents the total yield of the above four steps. The chemical and structural elucidation of eighteen compounds (**4a–g**, **4j**, **4t**, **5a–b**, **6a–b**, **6d–e**, **6i–j**, and **7a–b**) have been reported in our previous publication [17].

*2-Allyl-4-(benzo[d][1,3]dioxol-5-yl)phenol* (**4h**). Yield 40.0%; HPLC: 99.3%. ^1^H-NMR (CDCl_3_) δ 7.29–7.26 (m, 2H, 2- and 6′-H), 7.02–6.98 (m, 2H, 2′- and 6-H), 6.86–6.84 (m, 2H, 5- and 5′-H), 6.09–6.02 (m, 1H, -CH=CH_2_), 5.98 (s, 2H, -OCH_2_O-), 5.23–5.17 (m, 2H, -CH=CH_2_), 3.46 (d, 2H, *J* = 6.8 Hz, -CH_2_CH); HRMS [M + Na]^+^ calcd. 255.1021; found 255.1028.

*2-Allyl-4-(2,3-dihydrobenzo[b][1,4]dioxin-6-yl)phenol* (**4i**). Yield 68.0%; HPLC: 97.8%. ^1^H-NMR (CDCl_3_) δ 7.31–7.26 (m, 2H, 2- and 6-H), 7.06–7.01 (m, 2H, 2′- and 6′-H), 6.90 (d, 1H, *J* = 8.4 Hz, 5-H), 6.85 (d, 1H, *J* = 8.0 Hz, 5′-H), 6.10–6.00 (m, 1H, -CH=CH_2_), 5.23–5.17 (m, 2H, -CH_2_CH), 4.97 (s, 1H, -OH), 4.29 (s, 4H, -OCH_2_CH_2_O-), 3.46 (d, 2H, *J* = 6.4 Hz, -CH_2_CH); HRMS [M + H]^+^ calcd. 269.1178; found 269.1172.

*3-Allyl-2′-(trifluoromethyl)-[1,1′-biphenyl]-4-ol* (**4l**). Yield 43.0%; HPLC: 97.8%. ^1^H-NMR (CDCl_3_): δ 7.72 (d, 1H, *J* = 7.2 Hz, 6′-H), 7.53 (t, 1H, *J* = 7.2 Hz, 3′-H), 7.43 (t, 1H, *J* = 7.2 Hz, 5′-H), 7.32 (d, 1H, *J* = 7.2 Hz, 4′-H), 7.10–7.08 (m, 2H, 2- and 6-H), 6.84 (d, 1H, *J* = 8.0 Hz, 5-H), 6.09–5.99 (m, 1H, -CH=CH_2_), 5.19–5.15 (m, 2H, -CH=CH_2_), 3.44 (d, 2H, *J* = 6.0 Hz, -CH_2_CH); HRMS [M − H]^−^ calcd. 277.0840; found 277.0840.

*3-Allyl-3′-(trifluoromethyl)-[1,1′-biphenyl]-4-ol* (**4k**). Yield 59.8%; HPLC: 99.5%. ^1^H-NMR (CDCl_3_): δ 7.77 (s, 1H, 2′-H), 7.72–7.70 (m, 1H, 6′-H), 7.56–7.49 (m, 2H, 2- and 6-H), 7.39–7.35 (m, 2H, 3′- and 4′-H), 6.91 (d, 1H, *J* = 8.0 Hz, 5-H), 6.11–6.01 (m, 1H, -CH=CH_2_), 5.24–5.19 (m, 2H, -CH=CH_2_), 3.49 (d, 2H, *J* = 6.4 Hz, -CH_2_CH); HRMS [M + H]^+^ calcd. 301.0816; found 301.0820.

*3-Allyl-4′-(trifluoromethyl)-[1,1′-biphenyl]-4-ol* (**4l**). Yield 51.8%; HPLC: 98.9%. ^1^H-NMR (CDCl_3_): δ 7.67–7.62 (m, 4H, Ar′-H), 7.40–7.36 (m, 2H, 2- and 6-H), 6.92 (d, 1H, *J* = 8.0 Hz, 5-H), 6.10–6.01 (m, 1H, -CH=CH_2_), 5.24–5.19 (m, 2H, -CH=CH_2_), 3.49 (d, 2H, *J* = 6.0 Hz, -CH_2_CH); HRMS [M + H]^+^ calcd. 279.0997; found 279.0993.

*3-Allyl-2′,4′-bis(trifluoromethyl)-[1,1′-biphenyl]-4-ol* (**4m**). Yield 21.2%; HPLC: 98.7%. ^1^H-NMR (CDCl_3_): δ 7.90 (s, 1H, 3′-H), 7.38–7.35 (m, 2H, 2′- and 6′-H), 7.15–7.11 (m, 2H, 2- and 6-H), 6.95–6.81 (m, 1H, 5-H), 6.10–5.97 (m, 1H, -CH=CH_2_), 5.20–5.10 (m, 2H, -CH_2_CH), 3.48 (d, 2H, *J* = 6.4 Hz, -CH=CH_2_). HRMS [M + H]^+^ calcd. 347.0871; found 247.0869.

*3-Allyl-3′,5′-bis(trifluoromethyl)-[1,1′-biphenyl]-4-ol* (**4n**). Yield 41.2%; HPLC: 98.0%. ^1^H-NMR (CDCl_3_): δ 7.95 (s, 1H, 4′-H), 7.40–7.37 (m, 2H, 2′- and 6′-H), 7.15–7.11 (m, 1H, 2-H), 6.95–6.81 (m, 2H, 5- and 6-H), 6.12–6.00 (m, 1H, -CH=CH_2_), 5.23–5.14 (m, 2H, -CH_2_CH), 3.50 (d, 2H, *J* = 6.0 Hz, -CH=CH_2_). HRMS [M + H]^+^ calcd. 347.0871; found 347.0870.

*3-Allyl-4′-chloro-3′-(trifluoromethyl)-[1,1′-biphenyl]-4-ol* (**4o**). Yield 18.9%; HPLC: 97.5%. ^1^H-NMR (CDCl_3_): δ 7.93 (s, 1H, 3′-H), 7.41–7.36 (m, 2H, 2′- and 6′-H), 7.18–7.14 (m, 2H, 2- and 6-H), 7.05–7.01 (m, 1H, 5-H), 6.16–5.99 (m, 1H, -CH=CH_2_), 5.25–5.16 (m, 2H, -CH_2_CH), 3.54 (d, 2H, *J* = 6.4 Hz, -CH=CH_2_). HRMS [M + Na]^+^ calcd. 335.0426; found 335.0422.

*3-Allyl-4′-fluoro-[1,1′-biphenyl]-4-ol* (**4p**). Yield 23.9%; HPLC: 99.0%. ^1^H-NMR (CDCl_3_): δ 7.50–7.46 (m, 2H, 2- and 6-H), 7.32–7.30 (m, 2H, 2′- and 6′-H), 7.11–7.07 (m, 2H, 3′- and 5′-H), 6.88 (d, 1H, *J* = 8.4 Hz, 5-H), 6.11–6.00 (m, 1H, -CH=CH_2_), 5.23–5.18 (m, 2H, -CH=CH_2_), 5.05 (s, 1H, -OH), 3.47 (d, 2H, *J* = 4.8 Hz, -CH_2_CH); HRMS [M + H]^+^ calcd. 229.1029; found 229.1022.

*3-Allyl-3′,5′-difluoro-[1,1′-biphenyl]-4-ol* (**4q**). Yield 34.8%; HPLC: 98.6%. ^1^H-NMR (CDCl_3_): δ 7.35–7.31 (m, 2H, 2- and 6-H), 7.08–7.03 (m, 2H, 2′- and 6′-H), 6.89 (d, 1H, *J* = 8.0 Hz, 5-H), 6.76–6.69 (m, 1H, 4′-H), 6.10-5.97 (m, 1H, -CH=CH_2_), 5.24-5.18 (m, 2H, -CH=CH_2_), 5.07 (s, 1H,-OH), 3.48 (d, 2H, J = 6.0 Hz, -CH_2_CH); HRMS [M + H]^+^ calcd. 269.0754; found 269.0749.

*N-(3′-Allyl-4′-hydroxy-[1,1′-biphenyl]-3-yl)acetamide* (**4r**). Yield 56.9%; HPLC: 99.2%. ^1^H-NMR (DMSO-d_6_) δ 9.97 (s, 1H, -NH), 9.54 (s, 1H, -OH), 7.74 (s, 1H, 2′-H), 7.52 (d, 1H, *J* = 8.4 Hz, 4′-H), 7.32–7.28 (m, 2H, 2- and 6-H), 7.20 (d, 1H, *J* = 8.0 Hz, 5′-H), 6.89–6.87 (m, 1H, 5-H), 6.05–5.95 (m, 1H, -CH=CH_2_), 5.11–5.02 (m, 2H, -CH_2_CH), 3.36–3.34 (m, 2H, -CH=CH_2_), 2.05 (s, 3H, -CH_3_); HRMS [M + H]^+^ calcd. 268.1338; found 268.1347.

*N-(3′-Allyl-4′-hydroxy-[1,1′-biphenyl]-3-yl)methanesulfonamide* (**4s**). Yield 20.9%; HPLC: 99.7%. ^1^H- NMR (CDCl_3_): δ 7.41–7.33 (m, 5H, Ar-H), 7.18–7.16 (m, 1H, 2′-H), 6.89 (d, 1H, *J* = 8.0 Hz, 5-H), 6.74 (s, 1H, -NH), 6.05 (m, 1H, -CH=CH_2_), 5.22–5.17 (m, 2H, -CH_2_CH), 3.47 (d, 2H, *J* = 6.4 Hz, -CH=CH_2_), 3.04 (s, 3H, -CH_3_); HRMS [M + Na]^+^ calcd. 326.0827; found 326.0829.

#### 3.1.4. Syntheses of **5a–c** followed the general procedure

*3-(2-Methylbut-3-en-2-yl)-3′,5′-bis(trifluoromethyl)-[1,1′-biphenyl]-4-ol* (**5c**). Yield 19.5%; HPLC: 98.9%. ^1^H-NMR (CDCl_3_): δ 7.95 (s, 1H, 4′-H), 7.41–7.37 (m, 1H, Ar-H), 6.95–6.91 (m, 3H, Ar-H), 6.73 (d, 1H, *J* = 8.0 Hz, 5-H), 6.11–5.97 (m, 1H, -CH=CH_2_), 5.07 (s, 1H, -CH=CH_2_), 4.83 (s, 1H, -CH=CH_2_), 1.74 (s, 3H, -CH_3_), 1.71 (s, 3H, -CH_3_). HRMS [M + H]^+^ calcd. 378.1184; found 378.1180.

#### 3.1.5. Syntheses of **6a–j** Following Step III of the General Procedure

*3′-Allyl-3,4,4,5′-tetramethoxy-1,1′-biphenyl* (**6c**). Yield 97.5%; HPLC: 98.4%. ^1^H-NMR (CDCl_3_): δ 7.39–7.37 (dd, 1H, *J* = 8.4 Hz, *J* = 2.4 Hz, 2-H), 7.32 (d, 1H, *J* = 2.4 Hz, 6-H), 6.91 (d, 1H, *J* = 8.4 Hz, 5-H), 6.72 (s, 2H, 2′- and 6′-H), 6.08–5.98 (m, 1H, -CH=CH_2_), 5.12–5.05 (m, 2H, -CH=CH_2_), 3.93 (s, 6H, -CH_3_), 3.88 (s, 3H, -CH_3_), 3.87 (s, 3H, -CH_3_), 3.44 (d, 2H, *J* = 6.4 Hz, -CH_2_CH). HRMS [M + Na]^+^ calcd. 337.1416; found 337.1323.

*3-Allyl-4-(allyloxy)-3′,5′-bis(trifluoromethyl)-1,1′-biphenyl* (**6f**). Yield 95.8%; HPLC: 97.9%. ^1^H-NMR (CDCl_3_): δ 8.10 (s, 1H, 4′-H), 7.55–7.50 (m, 2H, 2′- and 6′-H), 7.35–7.31 (m, 1H, 2-H), 7.05–7.00 (m, 2H, 5- and 6-H), 6.15–6.00 (m, 2H, -CH=CH_2_ and -OCH_2_CH=CH_2_), 5.53–5.48 (m, 1H, -OCH_2_CH=CH_2_), 5.41–5.36 (m, 1H, -OCH_2_CH=CH_2_), 5.26–5.19 (m, 2H, -CH=CH_2_), 4.65 (d, 2H, *J* = 4.8 Hz, -OCH_2_CH=CH_2_), 3.54 (d, 2H, *J* = 6.4 Hz, -CH_2_CH); HRMS [M + H]^+^ calcd. 387.1184; found 387.1180.

*3-Allyl-4-((3-methylbut-2-en-1-yl)oxy)-3′,5′-bis(trifluoromethyl)-1,1′-biphenyl* (**6g**). Yield 89.5%; HPLC: 98.5%. ^1^H-NMR (CDCl_3_): δ 8.01 (s, 1H, 4′-H), 7.45–7.40 (m, 2H, 2′- and 6′-H), 7.35–7.30 (m, 1H, 2-H), 7.08–7.02 (m, 2H, 5- and 6-H), 6.05–5.97 (m, 2H, -CH=CH_2_ and -OCH_2_CH=CH_2_), 5.50–5.45 (m, 1H, -OCH_2_CH=CH_2_), 5.40–5.35 (m, 1H, -OCH_2_CH=CH_2_), 5.27–5.21 (m, 2H, -CH=CH_2_), 4.55 (d, 2H, *J* = 4.8 Hz, -OCH_2_CH=CH_2_), 3.59 (d, 2H, *J* = 6.4 Hz, -CH_2_CH), 1.56 (s, 3H, -CH_3_), 1.53 (s, 3H, -CH_3_); HRMS [M + H]^+^ calcd. 415.1497; found 415.1495.

*N-(3′-Allyl-4′-methoxy-[1,1′-biphenyl]-3-yl)acetamide* (**6h**). Yield 93.8%; HPLC: 99.5%. ^1^H-NMR (DMSO-d_6_): δ 9.97 (s, 1H, -NH), 9.54 (s, 1H, 2′-H), 7.74 (s, 1H, 2-H), 7.52 (d, 1H, *J* = 8.4 Hz, 5′-H), 7.33–7.28 (m, 2H, 6- and 6′-H), 7.20 (d, 1H, *J* = 8.0 Hz, 4′-H), 6.89–6.87 (m, 1H, 4-H), 6.05–5.95 (m, 1H, -CH=CH_2_), 5.11–5.02 (m, 2H, -CH=CH_2_), 3.86 (s, 3H, 4′-OCH_3_), 3.36–3.34 (m, 2H, -CH_2_CH), 2.05 (s, 3H, -COCH_3_); HRMS [M+Na]^+^ calcd. 304.1313; found 304.1298.

#### 3.1.6. Syntheses of **7a–d** Following Step III of the General Procedure

*3,5-Diallyl-3′,5′-bis(trifluoromethyl)-[1,1′-biphenyl]-4-ol* (**7c**). Yield 65.4%; HPLC: 98.4%. ^1^H-NMR (CDCl_3_): δ 8.12 (s, 1H, 4′-H), 7.51-7.46 (m, 2H, 2′- and 6′-H), 7.30-7.24 (m, 1H, 2-H), 7.05–7.01 (m, 2H, 5- and 6-H), 6.16–6.03 (m, 2H, -CH=CH_2_), 5.29–5.15 (m, 4H, -CH=CH_2_), 4.65–4.43 (m, 4H, -CH_2_CH); HRMS [M + H]^+^ calcd. 409.1003; found 409.0998.

*3-Allyl-5-(2-methylbut-3-en-2-yl)-3′,5′-bis(trifluoromethyl)-[1,1′-biphenyl]-4-ol* (**7d**). Yield 65.4%; HPLC: 97.4%. ^1^H-NMR (CDCl_3_): δ 8.09 (s, 1H, 4′-H), 7.48–7.44 (m, 2H, 2′- and 6′-H), 7.21–7.18 (m, 1H, 2-H), 7.10–7.06 (m, 2H, 5- and 6-H), 6.13–6.04 (m, 2H, -CH=CH_2_), 5.25–5.13 (m, 4H, -CH=CH_2_), 4.55–4.36 (m, 4H, -CH_2_CH), 1.66 (s, 3H, -CH_3_), 1.63 (s, 3H, -CH_3_); HRMS [M + H]^+^ calcd. 437.1316; found 437.1315.

## 4. Conclusions

To date, suppression of angiogenesis-dependent tumor growth has been a widely accepted strategy for cancer therapy. Although acquired drug resistance remains an insurmountable obstacle of tumor- targeting therapy, it is unlikely to occur or at least at a low rate if the genetically stable ECs are targeted. Thus, ECs have been proven to be an attractive and potent target for angiogenesis therapy [21]. In the present study, 37 allylated biphenols represented a novel biphenyl structural motif and possessed a unique mode of action in anti-angiogenic and anti-tumor activity. In detail, 3-(2-methylbut-3-en-2-yl)-3′,5′-bis(trifluoromethyl)-[1,1′-biphenyl]-4-ol (**5c**) showed the strongest inhibitory effects on the proliferation, migration and tube formation of HUVECs featuring anti-angiogenic properties, and its average anti-proliferative activities against four tumor cell lines (C26, Hela, A549, and HepG2) demonstrated that the biologically active allylated biphenol molecule has dual functions and the inhibitory effect of **5c** was specific on HUVEC migration and tube formation, rather than resulting from its cytotoxicity. 
